# *Staphylococcus aureus *biofilm formation at the physiologic glucose concentration depends on the *S. aureus *lineage

**DOI:** 10.1186/1471-2180-9-229

**Published:** 2009-10-28

**Authors:** Sander Croes, Ruud H Deurenberg, Marie-Louise L Boumans, Patrick S Beisser, Cees Neef, Ellen E Stobberingh

**Affiliations:** 1 Department of Medical Microbiology, Maastricht University Medical Center, Maastricht, The Netherlands; 2 Department of Clinical Pharmacy and Toxicology, Maastricht University Medical Center, Maastricht, The Netherlands

## Abstract

**Background:**

Since bacteria embedded in biofilms are far more difficult to eradicate than planktonic infections, it would be useful to know whether certain *Staphylococcus aureus *lineages are especially involved in strong biofilm formation. For this reason, *in vitro *biofilm formation of 228 clinical *S. aureus *isolates of distinct clonal lineages was investigated.

**Results:**

At 0.1% glucose, more than 60% of the *S. aureus *strains associated with multilocus sequence typing (MLST) clonal complex (CC)8 produced large amounts of biomass, compared to 0-7% for various other clonal lineages. Additionally, *S. aureus *bloodstream isolates associated with MLST CC8 and CC7 had similar biofilm forming capacities as their commensal counterparts. Furthermore, strong biofilm formation could not be attributed to a specific accessory gene regulator (*agr*) genotype, as suggested previously. The *agr *genotypes were strictly associated with the clonal lineages. Moreover, strong biofilm formation was not related to slime formation. Congo red agar (CRA) screening is therefore not useful as a qualitative screening method for biofilm formation.

**Conclusion:**

The adherence to polystyrene surfaces under physiologic glucose concentration (0.1%) was dependent on the clonal lineage. Strains associated with MLST CC8 were markedly more often classified as strong biofilm former at glucose concentrations of 0%, 0.1% and 0.25%.

The present study reveals that the MLST CC8 associated genetic background was a predisposing factor for strong biofilm formation *in vitro*, under all tested glucose concentrations.

## Background

One of the defense mechanisms of *Staphylococcus aureus *is the capacity to form biofilms. Bacteria embedded in biofilms are often difficult to eradicate with standard antibiotic regimens and inherently resistant to host immune responses [1,2]. As a result, treatment of many chronic *S. aureus *biofilm related infections, including endocarditis, osteomyelitis and indwelling medical device infections is hindered [3]. Biofilm formation is a multistep process, starting with transient adherence to a surface. Subsequently, specific bacterial adhesins, referred to as microbial surface components recognizing adhesive matrix molecules (MSCRAMMS) promote the actual attachment [4]. Next, during the accumulation phase, bacteria stick to each other and production of extracellular polymeric substances (EPS) and/or incorporation of host derived components, such as platelets, takes place, resulting in a mature biofilm. In circumstances of nutrient deprivation, or under heavy shear forces, detachment of bacteria appears through autonomous formation of autoinducing peptides (AIP) [5], with release and dispersal of bacteria as a consequence. It has been shown that expression of the accessory gene regulator (*agr*) locus, encoding a quorum-sensing system, results in expression of surfactant-like molecules, such as δ-toxin [6], contributing to the detachment.

Essential for biofilm development in *S. aureus *is the regulatory genetic locus staphylococcal accessory regulator (*sarA*), which controls the intracellular adhesin (*ica*) operon and *agr *regulated pathways [7]. It has been suggested that biofilm formation in methicillin-resistant *S. aureus *(MRSA) is predominantly regulated by surface adhesins, which are repressed under *agr *expression, while biofilm formation in methicillin-susceptible *S. aureus *(MSSA) is more dependent on cell to cell adhesion by the production of *icaADBC*-encoded polysaccharide intercellular adhesin (PIA), also referred as poly-*N*-acetylglucosamine (PNAG) or slime [8]. However a clear role for the *ica *locus of *S. aureus *is not as evident as that of *Staphylococcus epidermidis *[9].

In general, the presence of glucose represses the *agr *system through the generation of a low pH [10,11]. So far, biofilm development in physiologic glucose-supplemented medium (1 g/L), corresponding to normal blood glucose levels [12], has not been investigated. Biofilm formation often occurs on medical devices, like catheters and heart valves, which are in direct contact with normal (floating) blood. Furthermore, since it has been shown that the regulatory pathways for biofilm formation vary between strains [8], the question arose whether these strain-to-strain differences could be attributed to different clonal lineages.

The aim of the present study was to examine the contribution of the genetic background of both MRSA and MSSA to biofilm formation under physiologic glucose concentration. MRSA associated with the five major multilocus sequence typing (MLST) clonal complexes (CCs), i.e. CC5, CC8, CC22, CC30 and CC45 [13] and MSSA with the same MLST CCs, and also CC1, were included in this study, since it has been suggested that these lineages possess the ability to become MRSA [14]. The results were compared with those obtained with lineages normally not related to MRSA, i.e. CC7, CC12, CC15, CC25 and CC121 [15]. Furthermore, the aim was to evaluate whether slime production is indicative for strong biofilm formation in *S. aureus*.

## Results

### Characterization of the genetic background

The *spa *types and associated MLST CCs of all tested strains are shown in Table 1. The results of *spa *typing/BURP and MLST were in accordance for a representative set of 16 strains of each major *spa *type and associated MLST CC.

**Table 1:** Distribution of *spa *types and associated MLST CCs among *S. aureus *strains included in this study

associated MLST CC	ST	No. of MRSA strains	No. of MSSA strains	*agr *genotype	*spa *types MRSA strains (No.)	*spa *types MSSA strains (No.)
1	ST1	NA^#^	16	III	NA^#^	**t127 **(15), t1787
5	ST5/ST5	15	15	II	**t002 **(4), t003, t041, t045, t447 (8)	**t002 **(12), t179, t311, t2212
8	ST8/ST1411^a^	26	15	I	**t008 **(12), t052 (6), t064, t068 (5), t303, t622	**t008**^a ^(10), t190, t648, t701 (2), t2041
22	ST22/ST22	10	15	I	**t223 **(10)	**t005 **(9), t223, t474, t790, t1433, t1629, t2681
30	ST36/ST714^b^	10	15	III	**t012 **(7), t253 (2), t1820	t012 (2), **t021**^b ^(4), t238, t300, t318 (2), t438, t1130, t1504, t2572, t2854
45	ST45/ST45	11	15	I	**t038 **(8), t445 (2), t740	**t015 **(2), t026, t050, t065, t102, t230 (3), t583, t589, t620 (2), t772 (2)
7	ST7	-	15	I	-	**t091 **(15)
12	ST12	-	10	II	-	t060, t156 (2), **t160 **(5), t213, t771
15	ST15	-	15	II	-	**t084 **(11), t085, t491 (2), t1716
25	ST25	-	10	I	-	**t078 **(4), t081, t087, t258, t353, t1671, t1898
121	ST720^c^	-	15	IV	-	t159 (2), **t171**^c ^(4), t284, t408 (4), t645 (2), t659, t2213
Total		72	156			

# not available

**Boldface **indicates *spa *types on which multilocus sequence typing analysis was performed (ST, sequence type).

^a ^The strain *spa *typed as t008 had ST1411, a double locus variant of ST8 at the *gmk *and *tpi *locus.

^b ^The strain *spa *typed as t021 had ST714, a single locus variant of ST30 at the *arcc *locus.

^c ^The strain *spa *typed as t171 had ST720, a single locus variant of ST121 at the *yqil *locus.

### Phenotypic detection of slime producing ability onto Congo red agar

The different Congo red agar (CRA) screening methods described in the literature were evaluated [16-18]. The choice of the agar medium, either brain heart infusion or trypticase soy, did not influence the morphology. The majority of *S. aureus *strains (91%) displayed colonies with a normal morphology (smooth round colonies), indicating that most strains were low-slime producers. Without sucrose, all colonies were colored (bright) red to bordeaux red, irrespective of the agar medium used. Addition of sucrose to both agar media resulted in more dark colonies and made the dry crystalline morphology harder to recognize. With sucrose, all colonies on brain heart infusion agar with Congo red were colored red to bordeaux red, while strains on trypticase soy agar with Congo red displayed mostly purple to black colonies. Nuances in color were not corresponding to differences in morphology.

**Figure 1:**
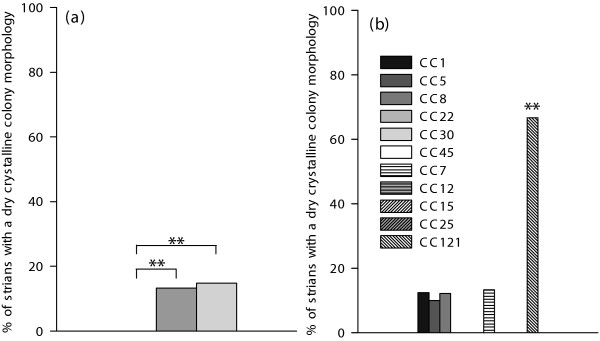
**Congo Red Agar screening of *S. aureus *isolates**. CRA screening for *S. aureus *with a dry crystalline colony morphology, which was considered indicative for slime formation. (a) The black bar (not visible, 0%) represents MRSA (*n *= 72), the dark grey bar represents MSSA with MRSA associated MLST CCs (*n *= 75) and the light grey bar represents MSSA with MSSA associated MLST CCs (*n *= 81). Asterisks denote statistically significant difference *P *< 0.01 (a) and statistically significant difference of individual CCs versus all other associated MLST CCs (b) *P *< 0.01.

MSSA strains showed more often a deviant, dry crystalline (rough) morphology (slime producing positive) than MRSA isolates, 14% (22 of 156) and 0%, respectively. A significant distinction in slime formation was observed between MRSA and MSSA with MSSA associated MLST CCs, i.e. CC7, CC12, CC15, CC25 and CC121, and with MRSA associated MLST CCs, i.e. CC1, CC5, CC8, CC22, CC30 and CC45 (*P *< 0.01), as shown in Figure 1a. MSSA associated with MLST CC121 had the highest prevalence of a deviant morphology, 67% (10 of 15) (Figure 1b).

### Detection of biofilm biomass with crystal violet staining

**Figure 2:**
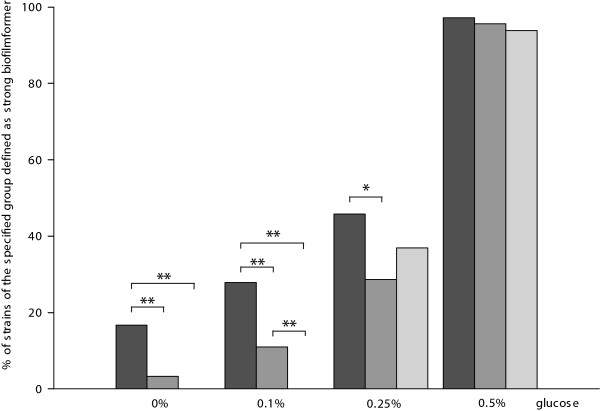
**Quantification of strong biofilm formation in MSSA and MRSA**. Quantification of strains of the specified group defined as strong biofilm former at different glucose concentrations. Black bars represent MRSA, dark grey bars represent MSSA with MRSA associated MLST CCs and light grey bars represent MSSA with MSSA associated MLST CCs. Asterisks denote statistically significant difference, (*) *P *< 0.05 and (**) *P *< 0.01.

**Figure 3:**
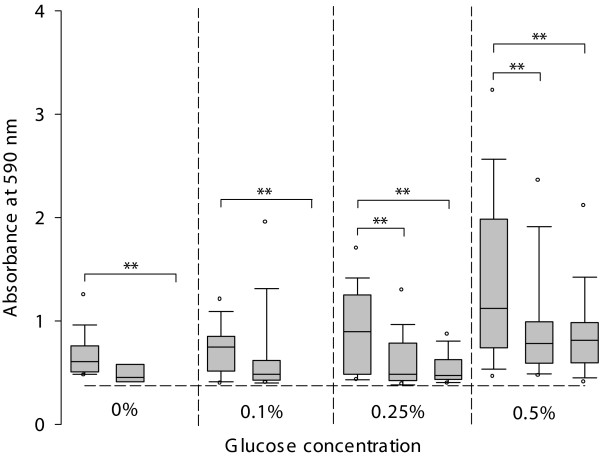
**Biomass quantification of MSSA and MRSA**. Absorbance (*A*_590_) of the crystal violet stained biofilm matrix for strong biofilm formers (with *A*_590 _above the threshold value of 0.374, represented by the horizontal dashed line) at different glucose concentrations. Boxplots at the left show MRSA, in the middle MSSA with MRSA associated MLST CCs and at the right MSSA with MSSA associated MLST CCs. The lower and higher boundary of the box indicates the 25^th ^and 75^th ^percentile, respectively. The line within the box marks the median. Whiskers above and below the box indicate the 90^th ^and 10^th ^percentiles. Open circles indicate the 95^th ^and 5^th ^percentiles. Asterisks denote statistically significant difference, (*) *P *< 0.05 and (**) *P *< 0.01.

**Figure 4:**
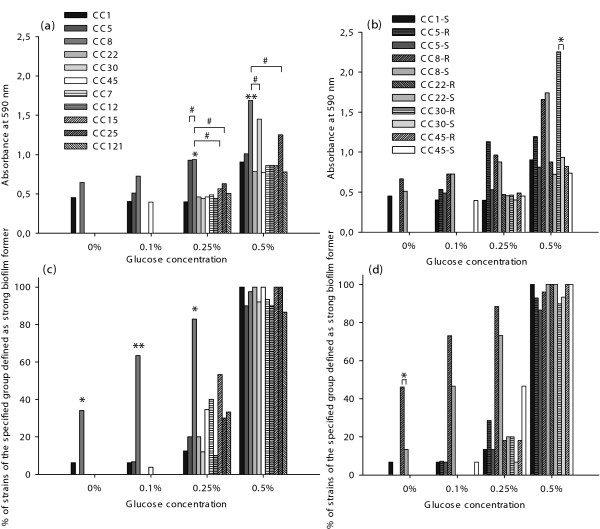
**Biomass formation related to the genetic background of *S. aureus***. Absorbance (*A*_590_) of the crystal violet stained biofilm matrix of strong biofilm forming *S. aureus *strains in relation to different associated MLST CCs (a) and of strong biofilm forming strains associated with MLST CC1, CC5, CC8, CC22, CC30 and CC45 (b). R in the legend represents MRSA and S represents MSSA. Quantification of strains of the specified genetic background defined as strong biofilm former at different glucose concentrations, (c) and (d). Asterisks denote statistically significant difference, (b) and (d), and statistical significant difference of individual CCs versus all other associated MLST CCs, (a) and (c), except #, (*) *P *< 0.05 and (**) *P *< 0.01.

Under physiologic glucose (0.1%) concentration, 13% (*n *= 30) of all strains formed a strong biofilm and all these strains were MRSA or had a MRSA associated MLST CC. MRSA and MSSA with MRSA associated MLST CCs, i.e. CC1, CC5, CC8, CC22, CC30 and CC45, were significantly more capable than MSSA with MSSA associated MLST CCs, i.e. CC7, CC12, CC15, CC25 and CC121, to form strong biofilms in the presence of 0.1% glucose (*P *< 0.01), but not at glucose concentrations of 0.25% and 0.5% (Figure 2). The higher the glucose concentration, the more strains produced biofilm above the *A*_590 _threshold value and were consequently classified as strong biofilm former. At glucose concentrations of 0.25% and 0.5%, the amount of biomass of the biofilms of strong biofilm forming strains was still significantly more for MRSA compared to MSSA irrespective of the MLST CCs (*P *< 0.01) (Figure 3). Of all strains classified as strong biofilm producers, MRSA and MSSA associated with MLST CC8 produced the most biomass under all tested glucose concentrations (Figure 4a and 4b). Strains defined as strong biofilm formers and associated with MLST CC5, CC25 and CC30 approached approximately the same level of biomass at the following glucose concentrations, i.e. CC5 at 0.25%, CC 25 at 0.5% and CC30 at 0.5% glucose, respectively.

The main contributors to the higher prevalence of MRSA and MSSA with MRSA associated MLST CCs to produce strong biofilms at 0.1% glucose were MLST CC8 isolates, approximately 60% (26 of 41), (Figure 4c), especially with a tendency towards MRSA (Figure 4d).

**Figure 5:**
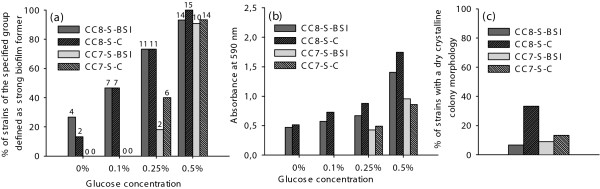
**Biofilm formation in *S. aureus *isolates of bloodstream infections and commensal origin**. Biofilm formation between *S. aureus *isolates of the same clonal lineage from blood stream infections (CC8 *n *= 15, CC7 *n *= 11) and of commensal origin (CC8 *n *= 15, CC7 *n *= 15), no significant differences were found (a). S in the legend represents MSSA, BSI represents bloodstream isolates and C represents commensal isolates. Number on each bar refers to number of isolates. Absorbance (A_590_) of the crystal violet stained biofilm matrix of strong biofilm formers at different glucose concentrations (b). CRA screening for colonies with a dry crystalline morphology (c).

Additionally, blood stream isolates of MSSA associated with MLST CC8 and MLST CC7 were included in the study, to address the question whether the isolation site is an (additional) predisposing factor for strong biofilm formation. MSSA associated with MLST CC7 are one of the main clonal lineages among blood stream isolates in our hospital [19]. No differences in the ability to produce strong biofilms were observed between bloodstream isolates and isolates of commensal origin among MSSA associated with MLST CC8 and CC7 (Figure 5a and 5b). Furthermore, no significant differences in slime-forming ability were observed (Figure 5c).

### Correlation between slime formation and development of biofilm biomass

In order to investigate whether slime production is indicative for strong biofilm formation, the correlation between these two characteristics was addressed. Phenotypic detection of slime production on CRA was not related to the quantitative detection of strong biofilms, measured by crystal violet staining, which was used as a gold standard. The sensitivity and specificity of the CRA method for *S. aureus *was approximately 9% and 90%, respectively (Table 2). Only a part of the slime producing strains surpassed the *A*_590 _threshold value for strong biofilm formation, namely 5%, 15%, 45% and 90% at 0%, 0.1%, 0.25 and 0.5% glucose, respectively.

**Table 2:** Correlation between slime formation (Congo red agar screening) and development of biofilm biomass (crystal violet staining).

Glucose	Sensitivity	Specificity	PPV	NPV	CRA+/CV+	CRA-/CV+	CRA+/CV-	CRA-/CV-
(%)	(%)	(%)	(%)	(%)	Number of *S. aureus *strains			
0	6.3	91.0	5.0	92.8	1	15	19	193
0.1	9.7	91.3	15.0	86.5	3	28	17	180
0.25	11.6	93.0	45.0	63.5	11	76	9	132
0.5	8.3	80.0	90.0	3.9	18	200	2	8

(PPV) positive predictive value (NPV) negative predictive value (CRA) Congo red agar screening (CV) crystal violet staining

### Distribution of *agr *types

Clonal lineages MLST CC7, CC8, CC22, CC25 and CC45 harbored *agr-*I, all CC5, CC12 and CC15 were characterized by *agr-*II, while all CC1 and CC30 were detected as *agr-*III. Furthermore, CC121 isolates carried *agr-*IV (Table 1). No consistent relationship was found between *agr *genotype and the ability to produce biofilm.

## Discussion

*In vitro *quantification of biofilm formation in distinct clonal lineages of *S. aureus *was performed to investigate whether there were differences in the capacity to form fully established biofilms. This study revealed that at 0.1% glucose, enhanced biofilm formation of *S. aureus *was strongly associated with MLST CC8 and observed in 60% of these isolates, while it varied between 0-7% for the other clonal lineages tested.

A higher percentage of MSSA (14%) than MRSA (0%) was found positive for slime producing ability, in concordance to the more important role of PIA/PNAG in MSSA than in MRSA biofilm development [8]. Addition of sucrose to CRA did not influence slime formation, suggesting that slime formation was carbohydrates independent. The results were consistent with previous findings in MRSA and MSSA isolates of O'Neill *et al*. In MSSA isolates increased *ica *expression and PIA/PNAG production (as determined with PIA/PNAG immunoblot) was correlated with 4% NaCl-induced biofilm formation, but not with glucose-induced biofilm production [8]. In addition, in MRSA, *ica *operon transcription was more potently activated by NaCl than by glucose, but did not result in PIA/PNAG formation [8]. Since it has recently been suggested that, in general, PIA/PNAG is a minor matrix component of *S. aureus *biofilms [5,9], and thus possibly hardly detectable by CRA screening, a low prevalence of slime producing strains was expected. Knobloch *et al*. and Mathur *et al*. reported a positive CRA assay result in only 4-5% of the *S. aureus *strains tested, in relative accordance with the results of this study, while Grinholc *et al*. mentioned 47% and 69% for MRSA and MSSA, respectively [16-18]. Jain *et al*. reported differences between blood stream isolates and commensal *S. aureus *isolates with regard to positive CRA screening, 75% and 20%, respectively [20]. The variations could be due to differences in genetic backgrounds of the strains used, or to differences in interpretation of the colonies. The definition of slime-forming strains used by Grinholc *et al*. and Jain *et al*. was based on the color of the colonies and not on the morphology. Furthermore, they both found a high consistency (96% and 91%, respectively) between CRA screening and biofilm biomass crystal violet staining [17,20]. In contrast, both in this study, as well in the studies by Knobloch *et al*., Rode *et al*., and Mathur *et al*. [16,18,21], no correlation was found between slime producing MRSA and MSSA isolates and an enhanced tendency to form large amounts of biomass. These studies strongly suggest that CRA screening forms no alternative for crystal violet staining to detect biofilm formation. Probably, the cell to cell adhesion, stimulated by the formation of PIA/PNAG, is less efficient than the expression of surface adhesins, in their contribution to produce more biomass.

As described before, the *agr *genotypes were strictly associated with the clonal lineages [22,23]. However, exceptions have been observed [24-27] which might be due to interstrain recombination and intrastrain rearrangements [28]. The association between *agr *genotypes and the genetic background explains the absence of a relationship between the enhanced ability to form biofilm and specific *agr *genotype(s). Although, there was a tendency that the *agr-*I genotype was associated with an enhanced capacity to form strong biofilms (data not shown), this was a reflection of the biofilm-forming capacity of strains associated with MLST CC8. In contrast to our results, Cafiso *et al*. described a link between *agr*-II genotype and the capacity to form strong biofilms, since all strains with *agr-*II genotype were associated with strong biofilm formation at 0.25% glucose. However, the genetic background was not taken into consideration [29]. Our findings revealed that strains associated with MLST CC5, CC12 and CC15 (all harboring *agr*-II) were classified as strong biofilm formers in only 21%, 9% and 53% of the cases at 0.25% glucose, respectively. Furthermore, the *agr*-II genotype encompass diverse strains, not including strains associated with MLST CC8 [22,23].

Biofilm formation was induced by increasing glucose concentrations up to 0.5% in both MRSA and MSSA isolates, which is entirely consistent with previously reported data [8,21]. However, MRSA produced significantly more biomass than MSSA with MSSA associated MLST CCs, under all tested glucose conditions. Especially strains associated with MLST CC8 contributed to this phenomenon. Furthermore, MSSA with MRSA associated MLST CCs were also capable to produce more biomass than MSSA with MSSA associated MLST CCs at 0.1% glucose. Variations in biofilm forming capacities in clonal lineages of *S. aureus *could be explained by unique combinations of surface-associated and regulatory genes [23] or by different expression levels of genes that regulate the different phases of biofilm formation. Since this study showed that the biofilm formation on polystyrene surfaces was the strongest for the MLST CC8 associated genetic background, further studies with other material or tissue are warranted. Recently, differences in adhesion to human airway epithelial cells have been observed between strains belonging to MLST CC8 and CC5, the latter demonstrating a generally lower adherence in both representatives of MRSA and MSSA [30]. An enhanced ability to adhere and invade these cells have also been shown for MRSA associated with the Brazilian/Hungarian clone, which belongs to MLST CC8 [15], compared to a population of MSSA with an unknown genetic background [31]. Furthermore, strains associated with the same clone were not included among our MLST CC8 isolates, but were previously classified as strong biofilm producers and designated superior in their ability to produce biofilm compared to isolates associated with the Pediatric clone (MLST CC5) [32].

To analyse possible other predisposing factors besides the MLST CC8 associated genetic background, bloodstream and commensal isolates of the same clonal lineage were compared. The biofilm forming capacity between MSSA bloodstream and commensal isolates, associated with MLST CC8 and CC7, was similar and consistent with the findings of Smith *et al*., who compared the biofilm forming capacity of Scottish clinical *S. aureus *strains collected from different isolation sites [33]. In contrast, Jain *et al*. described more frequently strong biofilm formers among *S. aureus *bloodstream isolates than commensal [20]. A possible explanation might be that all bloodstream isolates came from patients with peripheral intravenous devices, while this was not an inclusion criterion in the study by Smith *et al*. Peripheral or central line intraluminal colonization might be associated with strains that easily attach to (catheter) surfaces and as a consequence these strains could be dominant in leading to bloodstream infections.

## Conclusion

In summary, the present study reveals that the MLST CC8 associated genetic background was a predisposing factor for strong biofilm formation *in vitro*, under all tested glucose concentrations, i.e. 0%, 0.1%, 0.25% and 0.5%. At physiologic glucose concentration (0.1%), 0-7% of *S. aureus *from various clonal lineages were defined as strong biofilm former, compared to 60% for the *S. aureus *associated MLST CC8.

## Methods

### Bacterial strains

*S. aureus *strains (72 MRSA and 156 MSSA) investigated were isolated during 2005 to 2008 in the Maastricht University Medical Center, a tertiary 715-bed hospital, and originate from surveillance cultures (commensal flora) from individual patients, recovered from nasal swabs. MRSA and/or MSSA strains associated with MLST CC1, CC5, CC8, CC22, CC30, CC45, CC7, CC12, CC15, CC25 and CC121, were randomly selected from our institutional collection (Table 1). All MRSA strains were tested positive for the MRSA-specific *mecA *gene, by real-time PCR [34]. Additionally, 26 MSSA blood stream isolates from individual patients and associated with either MLST CC8 or CC7 were tested. These isolates were considered invasive.

### Characterization of the genetic background

Typing of the *spa *locus was carried out as described previously [19]. The *spa *types were assigned through the Ridom SpaServer http://spaserver.ridom.de and clustered into *spa*-CCs using the algorithm based upon repeat pattern (BURP) with Ridom StaphType 1.4 using the default settings [35,36]. Although, *spa *typing alone sometimes lacks discriminatory power, due to related *spa *repeat patterns within different clonal lineages and the emergence of homoplasies among *spa *sequences [37], it has been shown that *spa *typing/BURP results are often in agreement with results obtained by MLST [36,38]. Therefore, the associated MLST CCs were allocated through the SpaServer. To confirm the association between MLST and *spa *typing, in combination with BURP, MLST was performed on a representative set of 16 strains of each major *spa *type and associated MLST CC [39,40].

### Phenotypic detection of slime producing ability onto Congo red agar

MRSA (*n *= 72), MSSA with MRSA associated MLST CCs (*n *= 75), i.e. CC1, CC5, CC8, CC22, CC30 and CC45, and MSSA with MSSA associated MLST CCs (*n *= 81), i.e. CC7, CC12, CC15, CC25 and CC121, were cultured on Congo red agar (CRA) plates, either consisting of trypticase soy or brain heart infusion agar (both from Becton Dickinson) with 0.8 g/L Congo red (Prolabo, Leuven, Belgium) and without or with 5% sucrose (Merck, Darmstadt, Germany). Colony morphology and color were evaluated after incubation at 37°C for 24 h. Colonies with a dry crystalline (rough) morphology were considered deviant and slime producing positive [16], smooth round colonies were classified as low-slime producers.

### Detection of biofilm biomass with crystal violet staining

The polystyrene crystal violet adherence assay was carried out as described previously [41], with some modifications. Briefly, overnight cultures in Trypticase Soy Broth (TSB) without dextrose (Becton Dickinson, Le pont de Claix, France) were diluted until 10^8 ^CFU/mL in TSB containing 0%, 0.1%, 0.25% and 0.5% glucose. Individual wells of polystyrene, flat-bottomed 96-well plates (Greiner Bio-One, Frickenhausen, Germany) were filled with 100-μL aliquots of the cultures. As a negative control, uninoculated medium was used. *S. aureus *ATCC 25923 and one clinical *S. aureus *isolate from our collection, known to form fully established biofilms (*A*_590 _values within the highest range and stable) as observed during a pilot experiment, were added to each plate as reference standard [17] and positive control, respectively. After 4 h of adhesion at 37°C on a rocking platform at 25 oscillations min^-1^, the medium containing non-adhered cells, was replaced by 100 μL fresh broth and the plates were further incubated for 24 h. Next, the wells were washed three times with 200 μL 0.9% NaCl. Biofilms were fixed at 60°C during 1 h. Subsequently, 100 μl crystal violet solution (0.3% wt/vol) was added to all wells. After 15 min, the excess crystal violet was rinsed off by placing the plates under running tap water. Finally, after drying the plates, bound crystal violet was released by adding 100 μl 70% (vol/vol) ethanol with 10% isopropyl alcohol (vol/vol). Absorbance was measured spectrophotometrically at 590 nm (*A*_590_) and was proportional to biofilm biomass. All assays were performed in triplicate, and repeated on three occasions. The intra- and interday coefficients of variation for the assay were 14% and 23%, respectively. To obtain a threshold *A*_590 _value for which strong biofilm formation commences, the *A*_590 _values of all strains at the different glucose concentrations were sorted in ascending order and divided into quartiles. The distribution of *A*_590 _values in the lower three quartiles was similar at glucose concentrations of 0%, 0.1% and 0.25% and therefore used to determine the cut-off value (two standard deviations above the mean *A*_590 _value). The threshold *A*_590 _value was 0.374. Bacteria with *A*_590 _values above this value were considered strong biofilm formers.

### Determination of the *agr *type

The *agr *types were determined by a real-time multiplex PCR assay, as described previously [42].

### Statistical analysis

SPSS version 15.0 (SPSS Inc., Chicago, IL, USA) was used for statistical analyses. Chi-square analysis was used for comparison of the prevalence of strong biofilm formation or slime formation between the specified groups. Mann-Whitney U analysis was used to compare the *A*_590 _values between groups of strong biofilm formers. A *P *value of < 0.05 was considered to be statistically significant.

## Competing interests

The authors declare that they have no competing interests.

## Authors' contributions

SC carried out the biofilm measurement experiments and performed MLST, collected data and drafted the manuscript. RHD carried out the *spa *typing/BURP and participated in the design of the study. MLLB determined the *agr *types by a real-time multiplex PCR, helped with the statistical analysis and helped to write the manuscript. PB revised the manuscript critically. CN revised the manuscript critically. EES conceived of the study and participated in its design and coordination and helped to draft the manuscript. All authors read and approved the final manuscript
